# Rapamycin decreases DNA damage accumulation and enhances cell growth of WRN-deficient human fibroblasts

**DOI:** 10.1111/acel.12190

**Published:** 2014-02-05

**Authors:** Bidisha Saha, Alexander Cypro, George M Martin, Junko Oshima

**Affiliations:** Department of Pathology, University of WashingtonSeattle, WA, USA

**Keywords:** autophagy, DNA damage, mammalian target of rapamycin, rapamycin, Werner syndrome

## Abstract

Werner syndrome (WS), caused by mutations at the *WRN* helicase gene, is a progeroid syndrome characterized by multiple features consistent with accelerated aging. Aberrant double-strand DNA damage repair leads to genomic instability and reduced replicative lifespan of somatic cells. We observed increased autophagy in WRN knockdown cells; this was further increased by short-term rapamycin treatment. Long-term rapamycin treatment resulted in improved growth rate, reduced accumulation of DNA damage foci and improved nuclear morphology; autophagy markers were reduced to near-normal levels, possibly due to clearance of damaged proteins. These data suggest that protein aggregation plays a role in the development of WS phenotypes and that the mammalian target of rapamycin complex 1 pathway is a potential therapeutic target of WS.

Werner syndrome (WS) is a segmental progeroid syndrome caused by mutations in *WRN*, encoding a RecQ helicase/endonuclease (Oshima *et al*., 2013). WS cells display accumulation of DNA damage, accelerated telomere shortening, reduced replicative lifespan, and genomic instability (Rossi *et al*., 2010). The WRN protein participates in DNA damage repair, recombination, and replication (Rossi *et al*., 2010). The mammalian target of rapamycin (mTOR) pathway is a major growth regulatory pathway (Johnson *et al*., 2013). Rapamycin, a specific inhibitor of mTOR complex 1 (mTORC1), activates autophagy to facilitate degradation of cellular damaged protein aggregates and organelles to improve cellular function and extend lifespan (Johnson *et al*., 2013). Because of the significance of mTORC1 in aging (Johnson *et al*., 2013) and in DNA damage response (Hasty *et al*., 2013), we have begun to investigate the possible involvement of mTORC1 signaling in the pathogenesis of WS.

Our initial study using WS patient fibroblasts showed increased autophagy in primary and catalytic subunit of human telomerase (hTERT)-immortalized cells; these levels were further increased by rapamycin treatment (data not shown). Activation of basal autophagy in WS cells has been reported (Talaei *et al*., 2013). Because of wide variations among control cell lines, likely related to genetic backgrounds (data not shown), we generated human primary WRN knockdown fibroblasts using WRN shRNAs and selected the construct that maximally depleted the levels of WRN protein (15% residual protein) (Fig. 1A). The levels of the mature autophagosome marker, LC3-II, and of LC3-II/LC3-I ratios were increased in WRN knockdown cells as compared with a control using a scrambled shRNA (Fig. 1B,C). Twenty-four-hour treatment with 10 μm rapamycin suppressed mTORC1 signaling, as evidenced by the disappearance of phosphorylated ribosomal protein S6 (P-S6). LC3-II and the LC3-II/LC3-I ratio were both increased in WRN knockdown cells compared with untreated cells, indicating stimulation of autophagy (Fig. 1B,C). Rapamycin induced increases in LC3-II and decreases in p62, consistent with enhanced degradation of autophagosomes. These effects were greater in WRN knockdown cultures compared with the control (Fig. 1B,C). One micromolar rapamycin treatment gave similar results (data not shown). It is conceivable that increased autophagy may be a prosurvival mechanism in *WRN*-deficient cells, presumably a consequence of the accumulation of abnormal protein aggregates secondary to sustained DNA damage.

**Figure 1:**
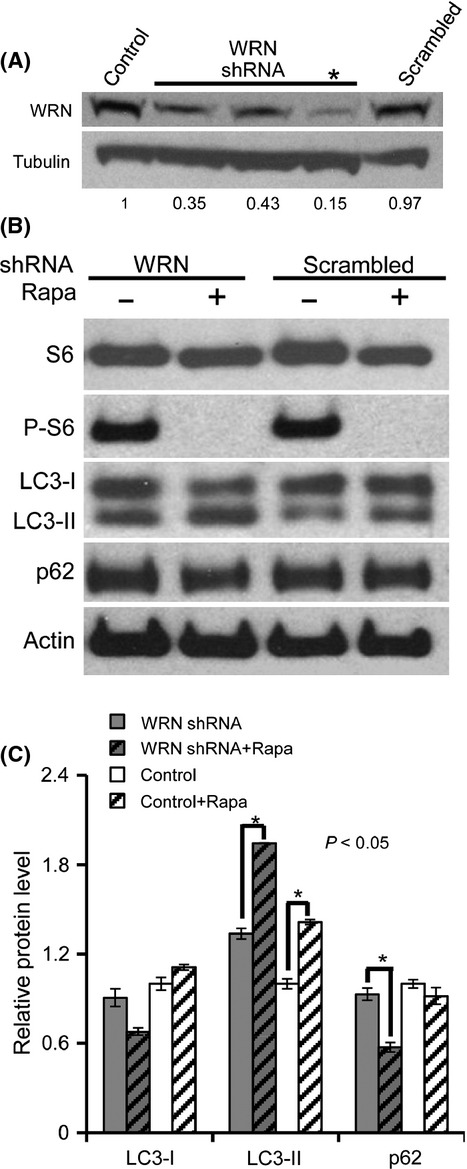
Increased autophagy and response to rapamycin in WRN knockdown primary fibroblasts. (A) Western analysis of WRN protein in normal fibroblasts, 82-6, treated with three different *WRN* (TRCN0000004899, TRCN0000004900, TRCN0000004903) or scrambled shRNAs; the relative WRN levels are normalized to tubulin levels. The shRNA (TRCN0000004903) with the lowest WRN level (*) was selected for further study. (B) Western analyses of S6, P-S6 LC3-I, LC3-II, p62, and β-actin in primary fibroblasts transfected with either WRN shRNA or scrambled shRNA with vehicle (DMSO) or rapamycin treatment (10 μm, 24 h). (C) Quantification of two independent western analyses in (B).

We examined the long-term effect of rapamycin treatment in WRN knockdown primary fibroblasts. After an initial slight drop in cell number, the growth rates began to increase in rapamycin-treated cultures compared with corresponding vehicle-treated cells for both scrambled shRNA control and WRN knockdown cells (Fig. 2A). At the end of 42 days, growth rates reached 1.3 population doubling (PD) per week for WRN knockdown cells with rapamycin compared with 0.4 PD per week without rapamycin. Comparable data for the controls were 1.2 PD per week and 1.0 PD per week, respectively. BrdU-labeling index was also higher in rapamycin-treated cultures: 48% for WRN knockdown cells with rapamycin compared with 25% without, and 72% for control with rapamycin compared with 58% without. The LC3-II and LC3-II/LC3-I ratio levels in untreated WRN knockdown cultures were higher than those of untreated controls, likely due to DNA damage accumulation. In rapamycin-treated WRN knockdown cells, the LC3-II and LC3-II/LC3-I ratios were reduced, reaching levels comparable with untreated controls (Fig. 2B,C). As expected, long-term rapamycin treatment led to reductions in LC3-II and LC3-II/LC3-I ratios in the controls. Expression levels of p62 were similar between WRN knockdown cells and controls, with decreased levels of p62 in rapamycin-treated cells (Fig. 2B,C). It has been shown that long-term rapamycin treatment results in the reduction in LC3-II following the clearance of protein aggregates (Spilman *et al*., 2010). Our data suggest long-term rapamycin treatment reduces protein aggregates in WRN knockdown cells by activating autophagy. DNA damage as assessed by p53-binding protein 1 (53BP1) foci was also significantly reduced in WRN knockdown cells (Fig. 2D,E). Moreover, long-term rapamycin treatment restored normal nuclear morphology of WRN knockdown fibroblasts (Fig. 2D). The average nuclear contour ratio was 0.78 ± 0.035 in scrambled shRNA controls; for WRN knockdown cells, it was 0.74 ± 0.089 (*P* = 0.033), reaching 0.81 ± 0.064 (*P* < 0.0001) after rapamycin treatment.

**Figure 2:**
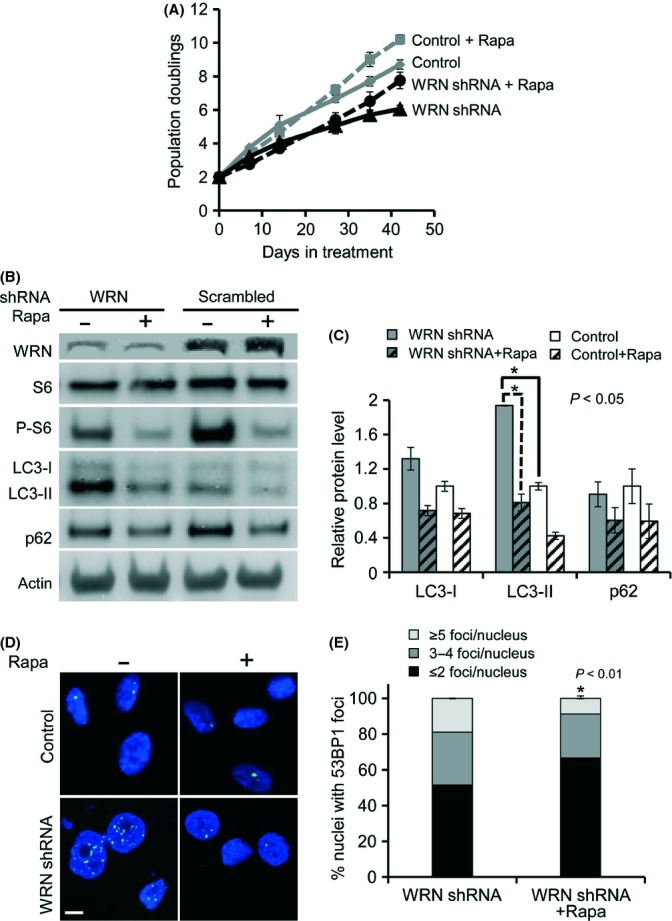
Long-term rapamycin treatment improves cell growth and reduces DNA damage in WRN knockdown cells. (A) Growth curves of fibroblasts with either WRN shRNA or scrambled shRNA (control) treated with either rapamycin (1 μm) or vehicle. Data are means ± SD of two independent experiments. (B) Western analyses of S6, P-S6 LC3-I, LC3-II, p62, and β-actin after 42 days of rapamycin treatment. (C) Quantification of two independent western analyses in (B). (D) Immunofluorescence detection of 53BP1 DNA damage foci in WRN shRNA and control cells after 40 days of rapamycin treatment. Cells were stained for 53BP1 (green) and DNA (using DAPI; blue). Scale bar = 10 μm. (E) Percentages of nuclei with variable numbers of 53BP1-labeled foci in vehicle- and rapamycin-treated cells. *P < 0.01.

DNA damage signaling modulates mTOR activity through p38/AKT as well as ATM/p53 (Hasty *et al*., 2013). Both the mTORC1-mediated growth stimulation and p53/p21-mediated cell cycle arrest may lead to cellular senescence (Demidenko & Blagosklonny, 2008). In WS cells, p38/p21 induces cell cycle arrest (Davis *et al*., 2005). Although off target effect of shRNA cannot yet be excluded, our results are consistent with the notion that accumulation of DNA damage led to increased protein aggregates that can compromise DNA repair processes and increase DNA damage (Bae & Guan, 2011). Rapamycin-mediated elimination of protein aggregates reduces its negative feedback, contributing to overall improved cellular function. Accumulation of insoluble protein aggregates and increased oxidative damage in WS cells have been reported by Talaei *et al*. (2013). H_2_S reduces protein aggregates and decreases oxidative damage (Talaei *et al*., 2013). The reduced DNA damage in rapamycin-treated WRN knockdown cells may also be due, in part, to accelerated replication; most of the accumulated DNA damage in WRN knockdown cells was shown to occur in nonreplicating cells (Szekely *et al*., 2005). Clearance of protein aggregate by rapamycin improves growth capacity of another progeroid syndrome, the Hutchinson–Gilford Progeria syndrome (Cao *et al*., 2011), and extends lifespan of *LMNA* knockout mice (Ramos *et al*., 2012). The present study now supports the potential therapeutic use of mTORC1 inhibitors in the treatment for WS.

## Supporting Information

**Data S1.** Experimental procedures.
