# Supplementary material for: Rapamycin decreases DNA damage accumulation and enhances cell growth of WRN-deficient human fibroblasts
Source: Aging Cell. 2014 Feb 5;13(3):573–5. doi: 10.1111/acel.12190 (PMC4032596; doi:10.1111/acel.12190)
Supplement: Supplementary file 1 — Data S1. Experimental procedures. [file acel0013-0573-sd1.docx]

**Supplemental Materials**

**Experimental Procedures**

**Cell lines and cell culture**

A human primary fibroblast, 82-6, was maintained under standard cultural condition as described previously (Saha *et al.* 2013). Cell numbers were counted at every passage using hemocytometers. Population doublings (PD) of the cultures were calculated as log 2 of the fold increase of cell numbers. For rapamycin treatment, fresh media containing rapamycin (R-5000, LC Laboratories, Woburn, MA) were applied to the fibroblasts. For long-term treatment, four cultures (WRN knockdown cells and control shRNA cells, with and without rapamycin) were initiated at the same time and fresh media containing 1 µM rapamycin (or vehicle only) were changed every other day.

**WRN shRNA-mediated depletion of WRN protein**

Five WRN-specific shRNAs (Clone ID: TRC0000004899, TRC0000004900, TRC0000004901, TRC0000004902, TRC0000004903) designed by The RNAi Consortium (TRC) were obtained from Thermoscientific (Lafayette, CO). The shRNAs were cloned into pLKO.1 lentiviral expression vector containing a human U6 promoter. The pLKO.1 expressing a scrambled shRNA (plasmid SHC202, non-targeting shRNA) (Sigma-Aldrich, St Louis, MO) was used as a control. The lentiviruses were packaged by co-transfecting human 293T cells with transfer vector pLKO.1 DNA, packaging plasmid psPAX2 and envelope plasmid pMD2G using FuGENE transfection reagent (Roche Applied Science, Indianapolis, IN) following manufacturer’s instructions. Viral supernatants were filtered through a 0.22-μm filter and stored at −80°C until use. 82-6 fibroblasts at passage 5 were transduced with either WRN or scrambled shRNA lentivirus for 48 hours. After 48 hours of transduction, cells were selected with puromycin (1 μg/ml) for 4 days. The levels of WRN protein was assessed by Western blot analysis after 10 days of shRNA transfection as described below. Three WRN shRNAs, TRCN0000004899, TRCN0000004900 and TRCN0000004903, which showed some reduction of WRN protein in hTERT fibroblasts, were further tested in primary fibroblasts (Fig 1A).

**BrdU incorporation assay**

To determine the fraction of proliferating cells, a BrdU incorporation assay was carried out using a 5-Bromo-2´-deoxyuridine (BrdU) labeling and detection kit I (11 296 736 001, Roche Applied Science, Indianapolis, IN) according to the manufacturer’s instructions as described before (Saha *et al.* 2013).

**Western blot analysis**

Western blot analyses were performed as described before (Saha *et al.* 2013). Two or ten micrograms of total protein isolated from primary fibroblasts were used for the study. The antibodies used in the study were rabbit anti-LC3 (1:1000, NB100-2331, Novus Biologicals, Littleton, CO), mouse anti-p62/SQSTM1 (1:2000, H00008878-M01, Abnova, Taipei, Taiwan), rabbit anti-P-S6 (Ser235/236) (1:2000, 2211, Cell Signaling Technology, Danvers, MA), rabbit anti-S6 (1:2000, 2217, Cell Signaling Technology), mouse anti-WRN (1:2000, W0393, Sigma Aldrich), mouse anti-β-actin (1:40,000, clone AC-15, Sigma-Aldrich) and mouse anti-β-tubulin (1:2500, T4026, Sigma-Aldrich). The secondary antibodies included biotinylated anti- mouse, anti-rabbit or anti-goat IgG (Vector Laboratories, Burlingame, CA, USA). The bands were quantified using Image J software (NIH Windows version) and normalized against β-actin or -β-tubulin levels.

**Immunofluorescence**

Immunofluorescenc was performed as described previously (Saha *et al.* 2013). The antibodies used were anti-53BP1 (1:100, NB100-304, Novus Biologicals) and secondary antibody, goat anti-rabbit Alexa Fluor 594 (1:200, Invitrogen Molecular Probes, Eugene, OR).

**Image processing and quantitative analysis of DNA damage foci**

The images were obtained in Z-series using the same exposure conditions with a Leica inverted microscope (Leica Microsystems, Buffalo Grove, IL) at the Keck Imaging Center (University of Washington, Seattle, WA). The images were deconvoluted using AutoQuant X software, version X3.0.1 (Media Cybernetics Inc., Bethesda, MD). The numbers of 53BP1 foci in each cell were counted after intensity based thresholding using Imaris software (Bitplane, South Windsore, CT). A minimum of 170 cells were counted for each condition.

**Nuclear morphology**

To assess the degree of nuclear irregularity, nuclear contour ratios (NCR, 4π x area/perimeter^2^) were measured in randomly selected nuclei of control and WRN knockdown fibroblasts using MetaMorph software as described previously (Saha et al., 2010). Approximately 120 cells were analyzed for each cell lines.

**Statistics**

Statistical significance was determined by the Student t-test.

**References**

Saha B, Zitnik G, Johnson S, Nguyen Q, Risques RA, Martin GM, Oshima J (2013). DNA damage accumulation and TRF2 degradation in atypical Werner syndrome fibroblasts with LMNA mutations. *Front Genet*. **4**, 129.

Saha B, Lessel D, Hisama FM. Leistritz DF, Friedrich K, Martin GM, Kubisch C, Oshim, J (2010). A Novel LMNA Mutation Causes Altered Nuclear Morphology and Symptoms of Familial Partial Lipodystrophy (Dunnigan Variety) with Progeroid Features. *Mol Syndromol* **1**, 127-32.
